# Gain-loss frequency and final outcome in the Soochow Gambling Task: A Reassessment

**DOI:** 10.1186/1744-9081-5-45

**Published:** 2009-11-09

**Authors:** Ching-Hung Lin, Yao-Chu Chiu, Jong-Tsun Huang

**Affiliations:** 1 Institute of Neuroscience, School of Life Sciences, National Yang-Ming University, Taipei, Taiwan, Republic of China; 2 Laboratory of Integrated Brain Research, Department of Medical Research & Education, Taipei Veterans General Hospital, Taipei, Taiwan, Republic of China; 3 Department of Psychology, Soochow University, Taipei, Taiwan, Republic of China; 4 Institute of Neural and Cognitive Sciences, China Medical University & Hospital, Taichung, Taiwan, Republic of China

## Abstract

**Background:**

Behavioral decision making literature suggests that decision makers are guided less by final outcome than by immediate gain-loss. However, studies of the Iowa Gambling Task (IGT) under dynamic and uncertain conditions reveal very different conclusions about the role of final outcome. Another research group designed a similar yet simpler game, the Soochow Gambling Task (SGT), which demonstrated that, in dynamic decision making, the effect of gain-loss frequency is more powerful than that of final outcome. Further study is needed to determine the precise effect of final outcome on decision makers. This experiment developed two modified SGTs to explore the effect of final outcome under the same gain-loss frequency context.

**Methods:**

Each version of the SGT was performed by twenty-four undergraduate Soochow University students. A large-value (± $200, ± $550 and ± $1050) and a small-value (± $100, ± $150 and ± $650) contrast of SGT were conducted to investigate the final outcome effect. The computerized SGT was launched to record and analyze the choices of the participants.

**Results:**

The results of both SGT versions consistently showed that the preferred decks A and B to decks C and D. Analysis of learning curves also indicated that, throughout the game, final outcome had a minimal effect on the choices of decision makers.

**Conclusion:**

Experimental results indicated that, in both the frequent-gain context and the frequent-loss context, final outcome has little effect on decision makers. Most decision makers are guided by gain-loss frequency but not by final outcome.

## Background

Traditional economic theory defines a rational economic decision as one intended to maximize monetary outcome [1]. Nevertheless, the behavioral decision literatures [2-6] generally agree that decision-making behavior is not dependent on final outcome (long-term outcome, future consequence, overall gain, and loss of stimuli in the long run). These findings challenge traditional economic notions such as expected value and expected utility.

In contrast with the questionnaire and thus descriptive gambling tasks analyzed in conventional behavioral decision studies, Bechara et al. [7,8] designed a dynamic four-card game that substantially differed from the traditional descriptive games. Subjects in this experience-based game had no knowledge of the immediate value, probability, or final outcome of the four choices. This game is the renowned Iowa gambling task (IGT), which has been applied in critical affective theory to test the Somatic Marker Hypothesis (SMH). The SMH [7-13] suggests that normal decision makers' choice pattern can be predicted by the final benefit in the IGT [7,13,14], which is designed to examine real-life decisions under uncertainty. In the IGT, advantageous decks C and D confer relatively small gains and losses than decks A and B do in each trial, and both decks achieve a positive final outcome (+$250) within an average of ten trials. Disadvantageous decks A and B have relatively large gains and losses in each trial and yield negative final outcomes (-$250) within an average of ten trials.

Damasio *et al.*[8,10,15] suggested that under conditions of uncertainty, if decision makers rely on somatic markers as indexed by skin conductance responses (SCRs) instead of relying on logical reasoning [14], they can gradually hunch the long-term benefit, i.e., choose more good decks (decks C and D) than bad decks (decks A and B) overall. However, damage to the somatic marker system can cause the decision maker to make irrational and risky choices. An example is the performance of subjects with ventromedial prefrontal lesions. These subjects preferred bad decks (decks A and B) rather than good decks (decks C and D), and their anticipatory SCRs (before card turning) did not significantly differ between good and bad decks [14,16-18].

The IGT is widely used not only for neurological and psychiatric assessment [19], but also to compare monetary decision making between economic models [13,20,21]. Clearly, the two different approaches, behavioral decision making (myopic to final outcome) and SMH (foresighted to final outcome), yield very different conclusions regarding the role of final outcome. Damasio and Bechara et al. adopted a traditional economic perspective (foresighted to final outcome) of final outcome in their investigation of this dynamic-uncertain game.

The two approaches also differ in their standpoint to decision guiding (immediate frame vs. long-term calculation), task context (certainty or risk vs. uncertainty), and task process (description-based vs. experience-based).

A variation of the IGT developed by Chiu *et al. *[22,23], namely, the Soochow gambling task (SGT), suggested that normal decision makers are insensitive to final benefit [24,25]. They also proposed that another controlling factor, namely, gain-loss frequency, can predict the decision making behavior in SGT and elucidate some inconsistent phenomenon in IGT [24,25]. Their results are inconsistent with the serial findings of the Iowa group but are consistent with the behavioral decision making literature [3,6,26,27]. Normal decision makers are usually guided by immediate gain-loss and are insensitive to final outcome.

**Figure 1:**
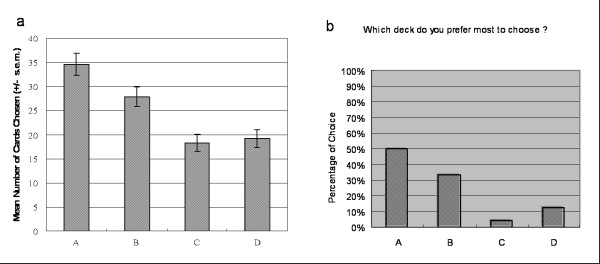
**Mean number of four-card selections in original SGT**. These figures were adopted from Chiu et al. 2005 [23]. (a) Normal decision makers preferred decks A and B, which had high frequency gain, but bad long-term outcome (EV). (b) The post-game questionnaire indicated that most subjects (20/24 subjects) preferred the bad EV decks in the concept stage. In the SGT, subjects were no longer implicitly guided by the EV and explicitly hunched the final outcome as suggested by Iowa group.

In SGT, decks A and B yield a bad long-term outcome (-$500) but a high-frequency gain (eight gains (A: +$200, B: +$100) two losses (A: -$1050, B: -$650)) in a block of ten trials. Decks C and D yield good final outcomes (+$500) but high-frequency losses (eight losses (C: -$200, D: -$100), two gains (C: +$1050, D: +$650)) in a block of ten trials (see Table 1). Chiu *et al. *found that decision makers prefer decks A and B to decks C and D under uncertainty. Most decision makers are insensitive to the final outcome not only implicitly but also explicitly, on a mental processing level [22,23] (see Figure 1). Gain-loss frequency had a stronger effect on choice behavior than final outcome did in the original SGT. Recently, this original finding of SGT was replicated by the Indiana university group [28] with a miniature value of SGT (original value of SGT divided by 100, e.g., $250/100 = $2.50).

**Table 1:** Gain-loss structure in original SGT.

Deck Card Sequence	A	B	C	D
1	200	100	**-200**	**-100**
2	200	100	**-200**	**-100**
3	200	100	**-200**	**-100**
4	200	100	**-200**	**-100**
5	**-1050**	**-650**	1050	650
6	200	100	**-200**	**-100**
7	200	100	**-200**	**-100**
8	200	100	**-200**	**-100**
9	200	100	**-200**	**-100**
10	**-1050**	**-650**	1050	650

**Final Outcome**	**-500 ($)**	**-500 ($)**	**500 ($)**	**500 ($)**

**Gain-loss Frequency**	**8 gains** **2 losses**	**8 gains** **2 losses**	**2 gains** **8 losses**	**2 gains** **8 losses**

**Figure 2:**
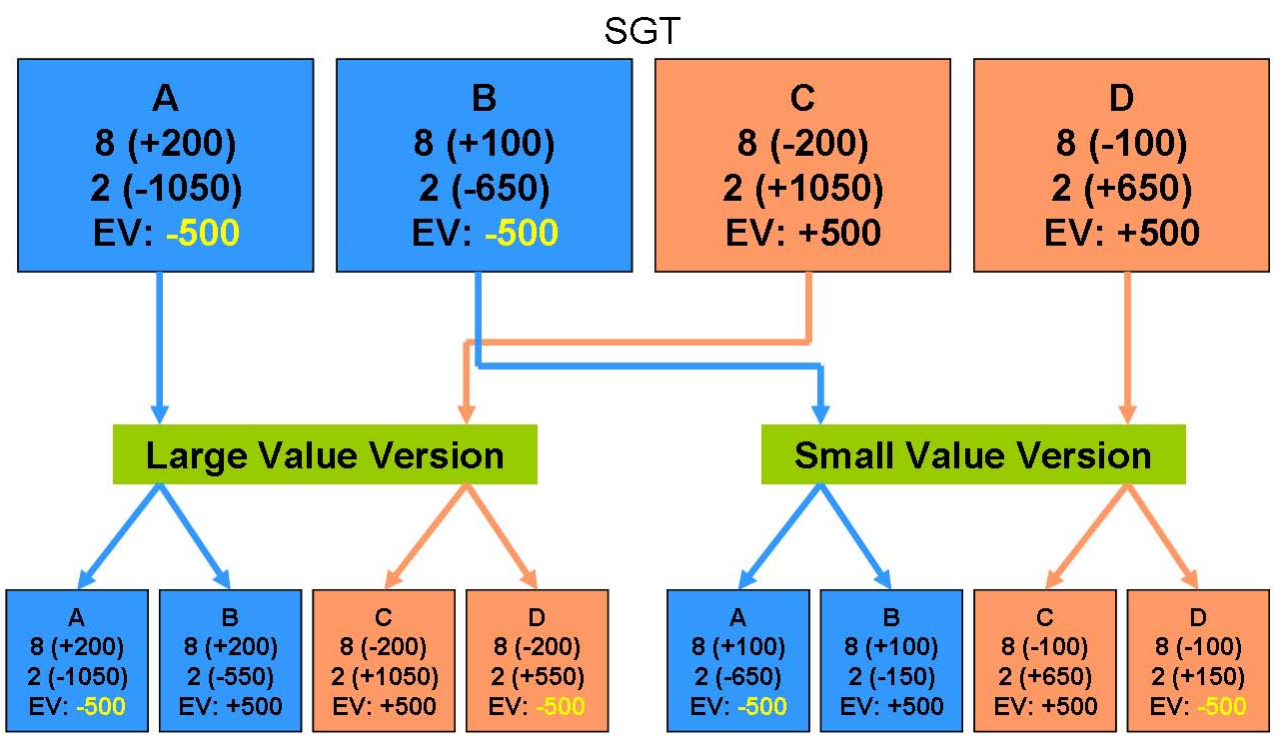
**Manipulation of the original SGT for further testing of EV**. In the original SGT, decks A and B exhibited high-frequency gain (eight gains, two losses) and negative EV (-$500), but decks A and B had different immediate values (+$200, -$1050 vs. +$100, -$650). However, decks C and D exhibited high-frequency loss (two gains, eight losses) and positive EV (+$500) whereas decks C and D had different immediate value (-$200, +$1050 vs. -$100, +$650). The present modified versions were generated by separating the immediate values obtained from decks A and C from those obtained from decks B and D. Large- and small-value versions were prepared to clarify the effect of EV and value contrasts.

Normal decision makers may be guided by gain-loss frequency rather than by final outcome. In the SGT context, final outcome may be a subordinate factor in predicting participants' behavior, but these results can not suggest accordingly that final outcome has less effect in guiding decision makers' choice. Although final outcome may be a subordinate predictor of behavior in SGT, the results indicate that it is still a major factor in decision making. A fair experimental manipulation in SGT is to evaluate the effect of final outcome by varying outcome under a constant gain-loss frequency, namely, by manipulating the final outcome variable under the same frequent-gain or frequent-loss context (Table 2 and Figure 2). To properly verify the effect of final outcome under the same frequency context, this study tested two versions of SGT. In both versions, decks A and B contained high-frequency gains (eight gains, two losses), and decks C and D contained high-frequency losses (two gains, eight losses). However, on average, decks A and D conferred bad final outcomes (-$500) and decks B and C conferred good final outcomes (+$500) after ten trials. To control for the contrast effect, the values in the first version (large value version) were ± $200, ± $550 and ± $1050, and those in the second version (small value version) were ± $100, ± $150 and ± $650 (Table 2). In both versions, participants who are insensitive to final outcome should have no preference between decks A (D) and B (C). However, if final outcome does significantly affect decision making under the same frequency context, decks B (C) would outperform decks A (D) in card selection. Moreover, the large- and small-value versions differed in value contrast (large value version: ± $200, ± $550 and ± $1050; small-value version: ± $100, ± $150 and ± $650). The large-value version situated the subjects in a relatively salient environment (large value contrast), and large losses (e.g., -$1050) were rare. Conversely, the small-value version situated the subjects in a relatively ambiguous environment (small-value contrast) and infrequent low-risk (small value) loss (e.g., -$650). If decision makers are easily influenced by the salient environment (large-value contrast), hunching the final outcome in the large-value version should be easier than in the small-value version. However, if subjects navigate easily in low-risk situations, hunching should be easier, and subjects should prefer the positive final outcome decks in the small-value version.

**Table 2:** Gain-loss structure in the two modified versions of SGT.

Large Value (± 200)	A	B	C	D	Small Value (± 100)	A	B	C	D
1	200	200	**-200**	**-200**	1	100	100	**-100**	**-100**
2	200	200	**-200**	**-200**	2	100	100	**-100**	**-100**
3	200	200	**-200**	**-200**	3	100	100	**-100**	**-100**
4	200	200	**-200**	**-200**	4	100	100	**-100**	**-100**
5	**-1050**	**-550**	1050	550	5	**-650**	**-150**	650	150
6	200	200	**-200**	**-200**	6	100	100	**-100**	**-100**
7	200	200	**-200**	**-200**	7	100	100	**-100**	**-100**
8	200	200	**-200**	**-200**	8	100	100	**-100**	**-100**
9	200	200	**-200**	**-200**	9	100	100	**-100**	**-100**
10	**-1050**	**-550**	1050	550	10	**-650**	**-150**	650	150

**Final Outcome**	**-500 ($)**	**500 ($)**	**500 ($)**	**-500 ($)**	**Final Outcome**	**-500 ($)**	**500 ($)**	**500 ($)**	**-500 ($)**

**Gain-loss Frequency**	**8 gains** **2 losses**	**8 gains** **2 losses**	**2 gains** **8 losses**	**2 gains** **8 losses**	**Gain-loss Frequency**	**8 gains** **2 losses**	**8 gains** **2 losses**	**2 gains** **8 losses**	**2 gains** **8 losses**

## Materials and methods

Forty-eight college students participated in this experiment. Each version was performed by twenty-four subjects (large-value version: ten males, thirteen females, and one subject for whom gender was not recorded; small-value version: eight males, sixteen females), and each subject completed one set of card arrangements (e.g., ABCD, ACDB, ADBC, etc.) to control for the position effect. According to the computerized version of the IGT [29], the SGT was programmed with Matlab 6.5 to record subject choices. In this experiment, most procedures were designed to follow the IGT administration procedure [7,14,17], except that participants were polled every ten trials after the first twenty trials to assess their subjective feelings and strategies for approaching this game [14]. The administration procedures included the original IGT instructions and facsimile money shown on the computer screen. Each gain or loss was summarized by bars in the top panel. Most subjects were initially unfamiliar with the internal gain-loss structure of gambles. Subjects were also told to earn as much money as possible or to lose as little money possible. However, none was apprised of the gamble structure until the end of the game. In the post-game questionnaire, the participants were asked to recall how many cards they had selected from each deck after 100 trials. The subjects were also asked to imagine a situation to replay the same game. They were then asked to write down the choice pattern over the four decks in this imaginary game.

## Results

**Figure 3:**
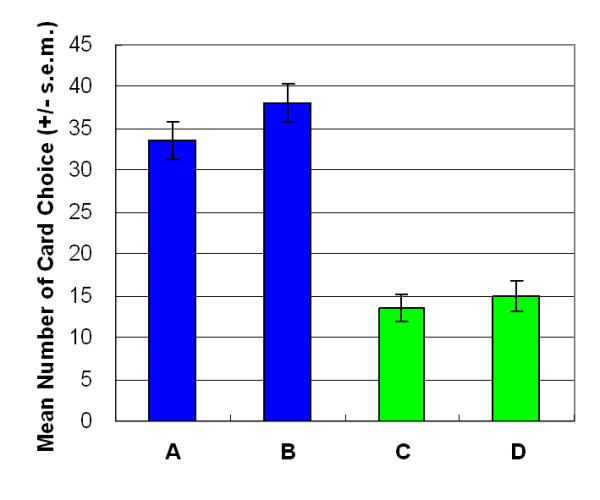
**Mean number of four-card selections in large-value version of SGT**. Participants preferred decks A and B to decks C and D. No significant effects were observed between decks A and B or between decks C and D. Gain-loss frequency had a far more powerful guiding effect. The EV did not significantly affect decision makers, even in the same gain-loss context.

**Figure 4:**
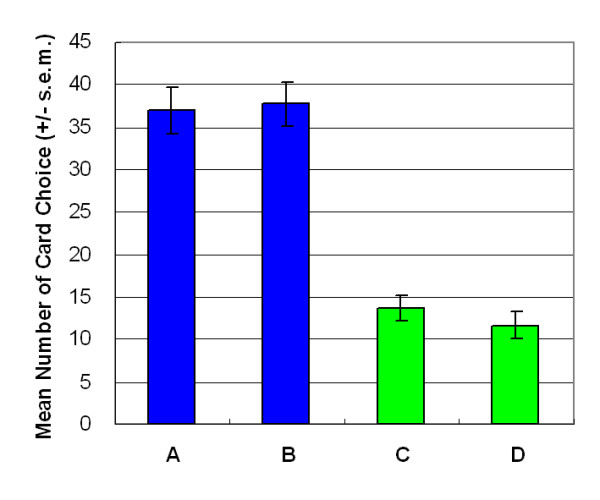
**Mean number of four-card selections in small-value version of SGT**. Even under varying monetary value (± $100, ± $150 and ± $650), the current version of SGT replicated the large-value version of SGT. Most subjects chose the high-frequency gain decks (A and B) rather than the high-frequency loss decks (C and D).

Empirical results indicated that participants made decisions based on gain-loss frequency rather than by final outcome. Most decision-makers preferred the high-frequency gain decks (A and B) to the high final outcome decks in both versions of SGT (Figures 3 and 4). In both versions, gain-loss frequency significantly differed between high-frequency gain decks (A, B) and high-frequency loss decks (C, D) (large-value version: *F *(1, 23) = 46.62, *p *< .01; small-value version: *F *(1, 23) = 73.99, *p *< .01). However, no statistically significant differences were observed in the final outcome dimension (large-value version: *F *(1, 23) = 3.15, *p *= .09; small-value version: *F *(1, 23) = 0.07, *p *= .80) or in the interaction between two factors (gain-loss frequency × final outcome) (large value version: *F *(1, 23) = 0.76, *p *= .39; small value version: *F *(1, 23) = .49, *p *= .49).

**Figure 5:**
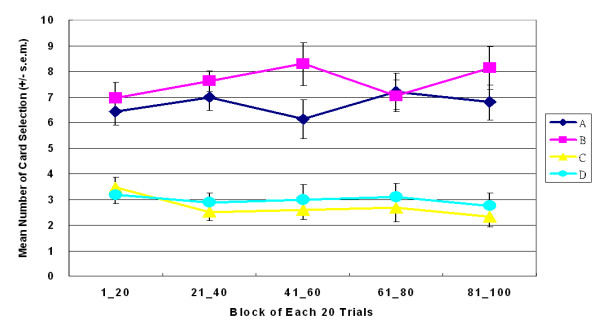
**Learning curve of five blocks in large-value version of SGT**. Each block consisted of twenty trials depicting preferences for four decks. The learning curves of four decks showed that frequent-gain decks A and B were preferred by normal decision makers from the beginning until the end of the game. No significant differences were observed between decks A and B or between decks C and D.

**Figure 6:**
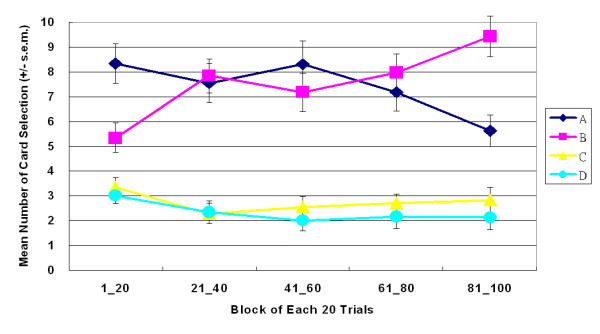
**Learning curves of five blocks in small-value version of SGT**. In this version of SGT, participants were preferred to choose frequent-gain decks A and B. Decks A (D) and B(C) had the opposite final outcome, but both decks showed the similar pattern of choices, which lasted until the end of the game. Decks A and B did not significantly differ, but the data revealed a crossover learning curve between decks A and B.

**Figure 7:**
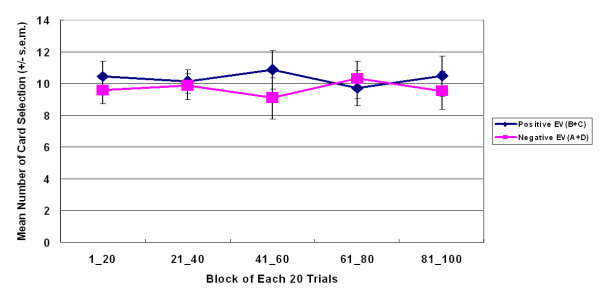
**Average of learning curves based on final outcome in large-value version of SGT**. Bad final outcome curve (A+D) was almost overlapped by the good final outcome curve (B+C). These statistical results showed that participants were insensitive to EV in these dynamic games even in the same gain-loss context.

**Figure 8:**
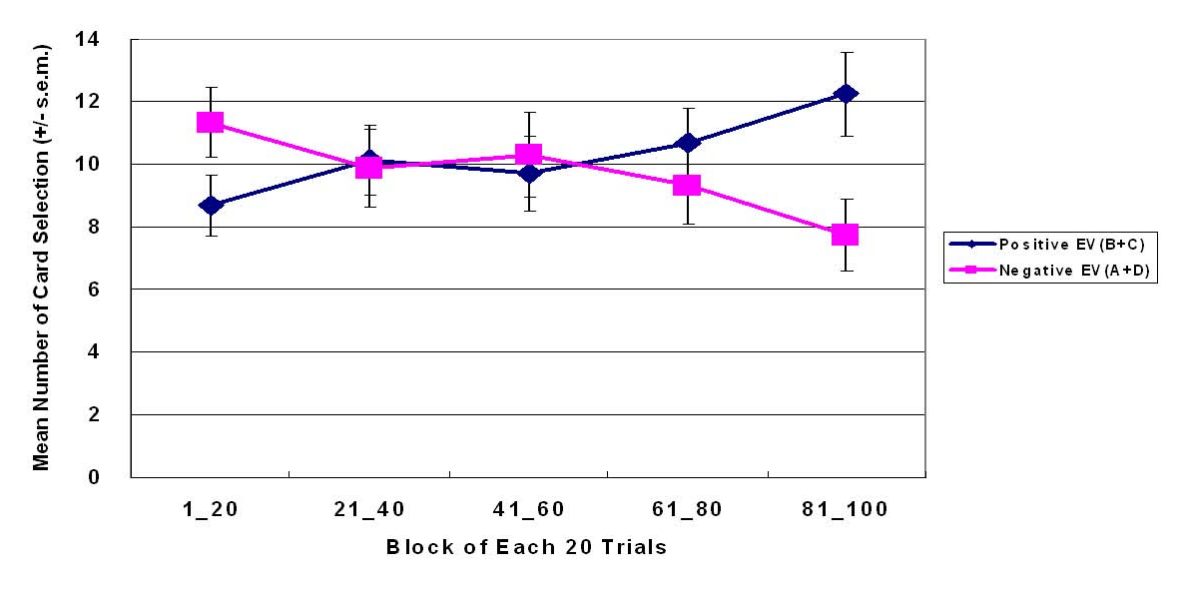
**Average learning curves based on final outcome in small-value version of SGT**. The final outcome curves of this version virtually replicated those of the large-value version. However, decision makers gradually shifted their preferences from the bad final outcome decks to the good final outcome decks. The learning effect was mainly contributed by decks A and B. This phenomenon indicates that final outcome may affect decision making in a relative low-risk context.

**Figure 9:**
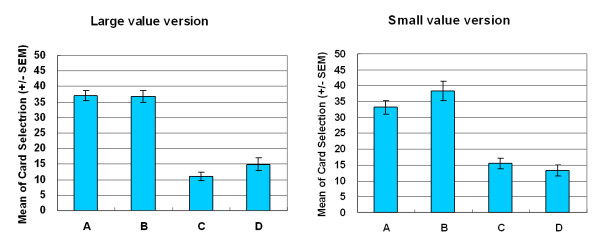
**Post-experimental memory assessment for both versions of SGT**. After the game, subjects were asked how many cards they chose from each of the four decks. Decks A and B were preferred to decks C and D.

**Figure 10:**
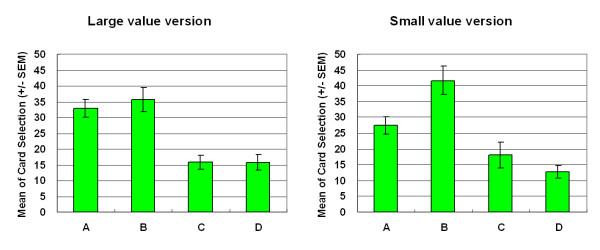
**Hypothetical assignment of 100 additional trials for both versions of SGT after the game**. Subjects were asked, "Suppose you were allowed to play the same game again. How would you allocate your choices for the four decks?" The response also showed that respondents would have favored decks A and B.

The learning curve indicated that, throughout the game, participants preferred decks A and B to decks C and D (Figures 5, 6). The learning curves for frequent-gain decks (A and B) were always above those of the frequent-loss decks (C and D). A three-factor (repeated measurement) ANOVA test of the learning curve was conducted. The five-block lines in Figures 5 and 6 show that, in both versions, decks A and B dominated the choices made by subjects. (large-value version: *F *(1, 23) = 46.62, *p *< .01; small-value version: *F *(1, 23) = 73.99, *p *< .01). In comparison, there are few significant main effects of final outcome (Figures 7 and 8) or interaction effects in both versions. Table 3 shows the statistical parameters. Notably, deck B was gradually preferred over deck A under the high-frequency gain context of small-value version (Figure 6). Nevertheless, the card selection curves of decks C and D were still far below those of decks A and B. No-cross over effect was observed across five blocks. The questionnaire survey results were consistent with the behavioral selection in both versions. In the memory evaluation, the participants correctly recalled that they chose decks A and B more frequently than they chose decks C and D in both versions (Figure 9). The subjects were then asked to imagine how they would allocate the choice pattern over the four decks if they are allowed to play the same game again. The hypothetical reallocation also showed that the participants preferred decks A and B to decks C and D (Figure 10).

**Table 3:** Statistical analysis (ANOVA) of learning curves in the two modified versions of SGT

Large value	F	Hypothesis df	Error df	p
FREQUENCY	46.62	1.00	23.00	**0.00**
OUTCOME	3.15	1.00	23.00	0.09
BLOCK	0.00	1.00	23.00	1.00
FRE * OUT	0.76	1.00	23.00	0.39
FRE * BLOCK	3.29	4.00	20.00	**0.03**
OUT * BLOCK	1.11	4.00	20.00	0.38
FRE * OUT * BLOCK	0.67	4.00	20.00	0.62

**Small value**	**F**	**Hypothesis df**	**Error df**	**p**

FREQUENCY	73.99	1.00	23.00	**0.00**
OUTCOME	0.07	1.00	23.00	0.80
BLOCK	147.68	2.00	22.00	**0.00**
FRE * OUT	0.49	1.00	23.00	0.49
FRE * BLOCK	3.64	4.00	20.00	**0.02**
OUT * BLOCK	2.41	4.00	20.00	0.08
FRE * OUT * BLOCK	3.21	4.00	20.00	**0.03**

## Discussion

This study showed that gain-loss frequency is the main guiding factor under uncertainty and that change in final outcome under the same frequency context does not significantly alter choice behavior. In both versions of the SGT, selections between decks A and B did not significantly differ from those between decks C and D. Normal decision makers were apparently attracted by the high-frequency gain decks (A and B) (Figures 5 and 6) and were unaware of the value of final outcome throughout the game (Figures 7 and 8). It is worth noting that according to the observation of Figures 6 and 8, the participants seem to be gradually sensitive to the change of final outcome between the two high-frequency gain decks A and B. The salient situation (large-value contrast) did not facilitate the ability to hunch the final outcome. However, the low-risk situation (small-value contrast) did give decision makers flexibility to explore the ambiguous environment. Therefore, the subjects could enter the hunch state relatively easily. This phenomenon implies that participants enter a hunch state when given useful cues in the relatively simple structure of the game but then change from an uncertainty state to a risky-decision state over time. The crossover learning curve between decks A and B in the small-value version remains to be identified. The data indicate that, in certain contrast contexts, when participants are still restraining in their choice over decks C and D (see Figures 3 and 4), they may have more time (trials) to compare the final benefit between decks A and B. The fact that decision makers were insensitive to final outcome or to EV (Expected Value) is consistent with the behavioral decision making literature [2,3,5,6,26,30]. Lichtenstein and Slovic [27] demonstrated that even the experimenter taught the decision makers about the information of final outcome, but this manipulation did not cause subjects to choose more on the higher final outcome selection. That is, the "EV inertia" rationality issue is very difficult to explain by traditional economic concepts. Although some experimental economists have acknowledged this phenomenon and have proposed solutions, the problem is still hotly debated between psychologists and economists [30].

However, the strong claim of SMH that is supported by bulky evidence from IGT, the Iowa group seems unnoticed of the EV inertia findings in decision literature. Instead, the SMH reconsidered the EV argument by introducing the interactive role of emotion. The SMH proposed that decision makers can foresee the benefit of final outcome under uncertainty with the help of emotion in an implicit way. The claim of SMH contradicts the findings of behavioral decision research. This claim also revolutionizes concepts of emotion in psychology and neuroscience. SMH corresponds to the basic concept of final outcome in traditional normative economics, which proposes that decision-makers act rationally to optimize their final outcome. Additionally, SMH proposes that emotional somatic markers, like SCRs, can help avoid bad choices so that long-term benefits can better be pursued under uncertainty.

Nevertheless, in a similar uncertainty context, both SGT versions demonstrate that the claim of the original SMH is unsupported in these uncertain and dynamic games. At both the implicit (Figures 3 and 4) and explicit (Figures 9 and 10) levels, decision-makers cannot precisely aggregate their gain-loss experience by value calculation across block trials. This study suggests that the somatic system resembles an internal bank within the human body. Its revenue and expenditure depend on frequency of gain and loss [19].

Above observations indicate that final outcome is hardly an accurate predictor in guiding card selection behavior. The results simply demonstrate the important role of gain-loss frequency in guiding decision behavior under uncertainty. Notably, studies of IGT and SGT [28,31] have all analyzed dynamic and uncertain situations, namely situations in which subjects were unaware of the internal structure of gambles.

Nevertheless, some studies [23,32] indicate that subjects gradually choose good decks and hunch the final outcome progressively in a relatively certain situation in which subjects can infer the gain-loss probability and value distribution. Therefore, contextual information may trigger different decision systems that guide choice behavior.

The frequency effect and probability learning [33] are commonly observed phenomena in various decision and animal studies [34-38]. The insensitivity to final outcome is observable not only in dynamic SGT games. Many decision studies have demonstrated similar phenomena in various gambles. For instance, Barron and Erev stated that the insensitivity of decision makers to final outcome is due to the underweighting of rare events [35,38].

Ahn *et al. *confirmed a frequency effect in IGT and the validity of the SGT for assessing behavioral level or modeling level [28]. They applied prospect theory [3,6,26] to explain how frequent small losses override infrequent large losses in the SGT. To obtain congruent predictions using IGT and SGT, Ahn *et al. *modified their "expectancy utility function" [39] with the "prospect theory" [3,26] after a reevaluation of SGT.

The dominance effect of gain-loss frequency in the IGT and SGT has also been reported under these dynamic-uncertain situations [40-42]. The present observation is consistent with numerous behavioral-analysis studies that suggested the schedule of reinforcement determines the pattern of choice behavior [43-46]. Some pioneering researchers [47,48] in behavioral analysis have illustrated that

"....the relation between a response and a later reinforcer contributes to responding only if no other reinforcers intervene; in other words, each reinforcer blocks responses that precede it from the effects of later reinforcers....." (Catania et al, 1988)

Like IGT and SGT, therefore, the relationship between each reinforcer (gain) and response (choice) in uncertain conditions is easily blocked and biased by other intervening stimuli and reinforcers (e.g., other decks, different values of gains or losses). This observation satisfactorily explains why the choice patterns of most subjects are dominated by immediate gain and loss: the causal relationship between the stimulus and reinforcement is difficult to conceptualize.

Further experimental investigations are needed to clarify both the insensitivity of final outcome and gain-loss frequency effect, particularly the concurrent schedule with reinforcement and punishments for decision making under uncertainty.

## Conclusion

The effect of gain-loss frequency was substantially larger than that of final outcome in both versions of SGT. The present observation correlates with numerous behavioral analysis studies [47] suggesting that the reinforcement schedule determines the pattern of choice behavior. This study demonstrates that subjects are insensitive to differences in final outcome given a similar (gain-loss) frequency context in both versions of SGT (large-value contrast and small-value contrast). Instead, subjects are significantly guided by gain-loss frequency under the same final outcome context. Most participants preferred high-frequency gain decks to high final outcome decks. However, it is worth noting that final outcome had a greater effect under the same context of high-frequency gains (A, B) in the small-value version. An intriguing issue requiring further study is decision making behavior under conditions other than uncertainty. Providing effective contextual information concerning the internal structure of the decision task may transform an uncertainty game into a risky choice task. Hopefully, future studies can further elucidate the relative contribution of gain-loss frequency and final outcome under the risk or certain situation as well as manipulating other value contrasts of immediate and long-term gain-loss.

## Abbreviations

EV: Expected Value; IGT: Iowa Gambling Task; SGT: Soochow Gambling Task; SMH: Somatic Marker Hypothesis.

## Competing interests

The authors certify that the above research findings are the best of our knowledge. The authors declare that they have no competing interests.

## Authors' contributions

CH, YC, and JT contributed to both versions of this work. CH and YC made equal contributions to analysis, experimental data collection, interpretation, and the initial drafting of the text. JT provided several reviews that were critical to reorganizing and revising the manuscript. All authors have approved the submitted version.
